# Gallbladder Interleukins in Children with Calculous Cholecystitis

**DOI:** 10.3390/pediatric13030054

**Published:** 2021-08-05

**Authors:** Arina Deņisova, Māra Pilmane, Arnis Eņģelis, Aigars Pētersons

**Affiliations:** 1 Institute of Anatomy and Anthropology, Riga Stradins University, Kronvalda Boulevard 9, LV-1010 Riga, Latvia; mara.pilmane@rsu.lv; 2 Department of Children Surgery, Riga Stradins University, Dzirciema street 16, LV-1007 Riga, Latvia; arnis.engelis@rsu.lv (A.E.); aigars.petersons@rsu.lv (A.P.)

**Keywords:** cholecystitis, gallbladder, children, interleukins, epithelium, connective tissue

## Abstract

Calculous cholecystitis connects to inflammation and various complications. It is a common disease in the paediatric population, yet it is still uncertain how inflammation factors are involved in its morphopathogenesis. Twenty calculous cholecystitis surgery tissue samples were obtained from 20 children. As a control, seven unaffected gallbladders were used. Tissues were immunohistochemically stained for IL-1α, IL-4, IL-6, IL-7, IL-8, IL-10, and IL-17A, and the slides were inspected by light microscopy. To evaluate statistical differences and correlations between interleukins, Mann–Whitney U and Spearman’s tests were used. Statistically significant difference between patient and control gallbladder epithelium was for IL-1α and IL-17A, but connective tissue—IL-1α, IL-4, IL-6, IL-7, IL-8, and IL-17A positive structures. A strong positive correlation in patients was detected between epithelial IL-1α and IL-1α in connective tissue, epithelial IL-6 and IL-7, IL-6 and IL-17A, IL-7 and IL-10, IL-7 and IL-17A, as well as between IL-6 and IL-7, IL-7 and IL-10 in connective tissue. The increase of IL-1α, IL-4, IL-6, IL-7, IL-8 and IL-17A positive structures suggests their role in the morphopathogenesis of calculous cholecystitis. The correlations between interleukins in epithelium and in connective tissues prove that the epithelial barrier function and inflammatory response in deeper layers are sustained through intercellular signalling pathways.

## 1. Introduction

Calculous cholecystitis is an inflammation of the gallbladder, that is caused by a long-standing cholelithiasis. Most of these patients have an asymptomatic presentation and biliary colic develops in 1 to 4% of these patients every year, whereas acute calculous cholecystitis develops in 20% of symptomatic patients [1]. Yet, it is not only the adults that are affected by this disease—the prevalence of calculous cholecystitis in children is as high as 1.9% to 4% [2]. A study that was made by Khoo et al. also showed that the incidence of cholecystectomy performed on children had been increased by three times in the period from 1997 to 2012 [3]. The main cause for cholelithiasis in children is increasing childhood obesity [2]. Haemolytic disease and hemoglobinopathies are no longer the primary risk factors for developing gallstones, especially in children [4].

The pathogenetic mechanism in calculous cholecystitis involves obstruction of the cystic duct by the gallstones that accumulate in the gallbladder. As a result, larger gallstones irritate the wall of the gallbladder, inducing an immune reaction [5]. Being a disease characterized by an inflammation, calculous cholecystitis is associated with immune cell migration and cytokine release. Yet, the information about cellular signalling through the use of interleukins is quite scarce. Previous studies have indicated that IL-1α, IL-4, IL-6, IL-7, IL-8, IL-10 and IL-17 are all expressed in adult gallbladder tissues due to various conditions [6,7,8,9,10,11,12]. Yet, the morphopathogenesis of cholecystitis and its interleukin profile in paediatric population is yet to be understood.

IL-1 family is produced by various cells in the human body, such as macrophages, dendritic cells, B lymphocytes, and epithelial cells [13]. Production of IL-1α is not only associated with an immune response to pathogens, but also with various physiological events, such as oxidative stress and hormonal stimulation. In the body, this interleukin acts as a pyrogen and inducer of acute phase response [14]. A rise in IL-1α levels is associated with gallbladder disease in menopausal women [6].

IL-4, a glycoprotein, which is produced by immune cells, is responsible for B cell activation and immunoglobulin synthesis. It also induces proliferation of hematopoietic, muscular, endothelial, and neuronal cells [15,16]. IL-4 deficiency in mice predisposes them to gallstone formation [17]. IL-4 also has anti-inflammatory properties, and it has been previously shown that IL-4 concentration in patients with gallstones is lower than in healthy individuals [7].

IL-6 is produced in infectious lesions by macrophages and monocytes, but it can also be produced by fibroblasts and endothelial cells [18]. IL-6 leads to acute phase protein and albumin synthesis, as well as induces cytotoxic T-cell differentiation and antibody production by B cells [19]. It has been revealed that IL-6 elevation in gallbladder tissues is related to inflammation and gallstone formation [7]. Another study made by Liu et al. demonstrated that IL-6 concentration was increased in patients with gallstones [8].

IL-7, an interleukin required in adaptive immune response, by regulating the development of mature B and T cells, is primarily expressed in stromal cells of lymphoid organs [20]. It also boosts the survival of naïve and memory T cells in the periphery, such as in intestinal mucosa of mice, where it is produced by epitheliocytes [21]. IL-7 is also found in inflammatory response to gallstone disease and in gallbladder carcinoma [9]. 

IL-8 is a chemoattractant for neutrophil leukocytes. It is produced by endotheliocytes, macrophages, and epithelial cells [22]. It is also needed for angiogenesis by inducing endothelial cell chemotaxis and proliferation [23]. There is a correlation between elevated IL-8 levels and primary sclerosing cholangitis, where it induces proliferation of cholangiocytes and has pro-fibrotic activity [10]. IL-8 also plays role in development of acute cholecystitis in the gallbladder wall [11]. 

IL-10 is a cytokine with both pro and anti-inflammatory properties. It is produced by monocytes and B cells, and it can promote B cell survival and antibody production [24]. IL-10 inhibits actions of other cytokines, such as IL-8. It can also inhibit monocyte MHC molecule expression, which can help in downregulation of excessive inflammation [22]. Increased IL-10 has been proven to heighten the risk of developing gallstones [8].

IL-17 family consists of six interleukins that are produced by Th17 and Tc cells [25]. IL-17 is responsible for T cell and macrophage activation, neutrophil leukocyte mobilization and upregulation of proinflammatory mediator expression [26]. IL-17 has been proven to be decreased in patients with cholelithiasis [7]. IL-17 is also associated with chronic inflammation of the bile ducts in patients with primary biliary cirrhosis as well as with biliary innate immunity [12].

Our aim was to research the appearance and distribution of different interleukins in calculous cholecystitis affected gallbladder wall in children.

## 2. Materials and Methods

### 2.1. Material Characteristics of Subjects

A total number of 20 calculous cholecystitis surgery tissue samples were obtained from 14 girls and 6 boys, aged 4 to 17 years. The inclusion criteria were a diagnosis of cholelithiasis, chronic pain syndrome, gastroenterologist’s confirmation that the pain is related to gallbladder and that further drug therapy is inappropriate, as well as patient and parental consent for surgery. The exclusion criteria were the diagnosis of cholelithiasis without pain syndrome, gastroenterologist’s opinion that drug therapy is still possible, and no consent of the patient and parents for the operation. The control group included material from autopsies of children from accidents (i.e., cause of death was mechanical trauma) and without gallbladder inflammation in the history. The patients were 5 boys and 2 girls aged 9 to 17 years. The obtained tissue samples were 2–3 mm^2^ in size, covering full wall of the gallbladder in region between fundus and neck. All of the tissues were referred for investigation to the Institute of Anatomy and Anthropology of Riga Stradins University by the Department of Children Surgery of the Children’s University hospital in 2007. The research was done in accordance with Helsinki declaration. The study was approved by Ethical committee at Riga Stradins University, the permit was issued on 10 May 2007.

### 2.2. Immunohistochemical Analysis

The tissues were fixated for 24 h in a mixture of 2% formaldehyde, 0.2% picric acid in 0.1 M phosphate buffer that had a pH of 7.2. Tissue samples were rinsed in Tyrode buffer, that contained 10% saccharose, for 12 h, then they were embedded into paraffin and later cut in 5 µm sections. For the general morphological evaluation of the wall of the gallbladder routine staining with haematoxylin and eosin was performed. IHC labelling was achieved with the use of the standard Biotin—Streptavidin method to detect: IL-1α (orb308737, working dilution 1:100, Biorbyt Limited, Cambridge, UK), IL-4 (orb10908, working dilution 1:100, Biorbyt Limited, Cambridge, UK), IL-6 (ab216492, working dilution 1:100, Abcam, Cambridge, UK), IL-7 (orb13506, working dilution 1:100, Biorbyt Limited, Cambridge, UK), IL-8 (orb39299, working dilution 1:100, Biorbyt Limited, Cambridge, UK), IL-10 (orb100193, working dilution 1:600, Biorbyt Limited, Cambridge, UK), and IL-17A (orb48920, working dilution 1:200, Biorbyt Limited, Cambridge, UK) [27,28].

The sample slides were analysed by light microscopy, whilst using non-parametric evaluation, which includes grading of positively stained cells in connective tissues and epithelium of the gallbladder in the visual field [29]. The results were labelled as follows: 0—no positive cells, 00/+—scant number of positive cells, 0/+—occasional positive cells, +—few positive cells, +/++—few to moderate number of positive cells, ++—moderate number of positive cells, ++/+++—moderate to numerous positive cells, +++—numerous positive cells in the visual field.

### 2.3. Statistical Analysis

The obtained data was processed using IBM SPSS software with 26.0 version (IBM company, North Castle, Armonk, NY, USA). To analyse the difference between patient and control groups, Mann–Whitney U test was performed, and the level of significance was 5%, thus the *p*-value was <0.05. To evaluate the results, Spearman’s rank correlation coefficient was used, where R < 0.2 indicated a very weak correlation, R = 0.2–0.4 a weak correlation, R = 0.4–0.6 a moderate correlation, R = 0.6–0.8 a strong correlation, but R = 0.8–1 a very strong correlation. 

## 3. Results

**Figure 1:**
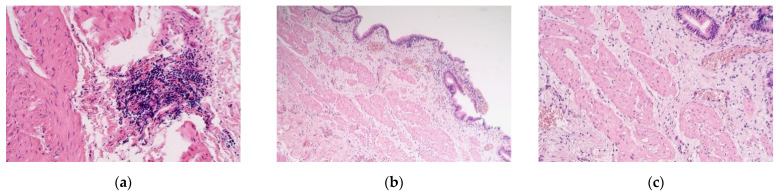
(**a**–**c**) Micrographs of gallbladder structures in children with cholecystitis. (**a**) Aggregation of lymphocytes in the muscle layer of the gallbladder. Haematoxylin and eosin, ×250; (**b**) the subepithelium infiltrated by the inflammatory cells of the gallbladder. Haematoxylin and eosin, ×100; (**c**) note the vacuolization of the smooth muscle cells. Haematoxylin and eosin, ×200.

The obtained tissue samples contained the whole gallbladder wall. Staining with Haematoxylin and eosin revealed muscular vacuolisation, vascular obliteration, patchy neoangiogenesis, inflammation, and even lymphoid aggregations (Figure 1a–c). 

**Figure 2:**
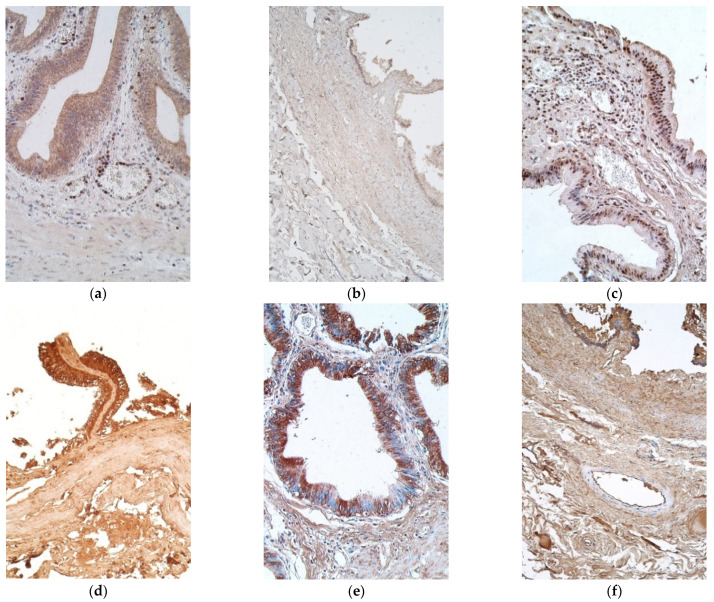
Micrographs of gallbladder in children with cholecystitis and in controls. (**a**) Note a moderate to numerous number of IL-1α positive epithelial cells along the positive inflammatory cells in the capillaries and subepithelial tissue. IL-1α IMH, ×200; (**b**) no IL-1α positive cells were detected in the control. IL-1α IMH, ×200; (**c**) a moderate number of IL-4 positive cells in the connective tissue. IL-4 IMH, ×200; (**d**) note numerous IL-4 positive epitheliocytes and a lack of IL-4 positive cells in the connective tissues of control. IL-4 IMH, ×200; (**e**) numerous IL-6 containing epitheliocytes in a patient. IL-6 IMH, ×200; (f) control samples with also numerous IL-6 positive epithelial cells. IL-6 IMH, ×200.

Interleukin positive cells were observed more in epithelium, except with IL-8 in patient samples and IL-1α in the controls (Table 1 and Table 2). IL-1α positive cells were seen in a moderate number in the epithelium of patient samples, while in connective tissue there were only a few to moderate number of positive cells (Figure 2a). On the contrary, control samples contained no positive cells in the epithelium and connective tissue, with the exception of two samples of the connective tissues, where there were a scant number and few positive cells (Figure 2b). 

IL-4 marked from moderate to numerous positive cells in epithelium of patients, whilst control samples had numerous number of such epitheliocytes. Patient samples also had mostly moderate number of positive cells in connective tissues, whilst controls showed only a few, with one sample containing none (Figure 2c,d).

Moderate to numerous IL-6 positive epitheliocytes were found in the patient samples, whilst the controls demonstrated numerous positive cells in epithelium. Patient tissues also had a moderate number of positive cells in the connective tissues, but controls possessed only a few (Figure 2e,f).

**Figure 3:**
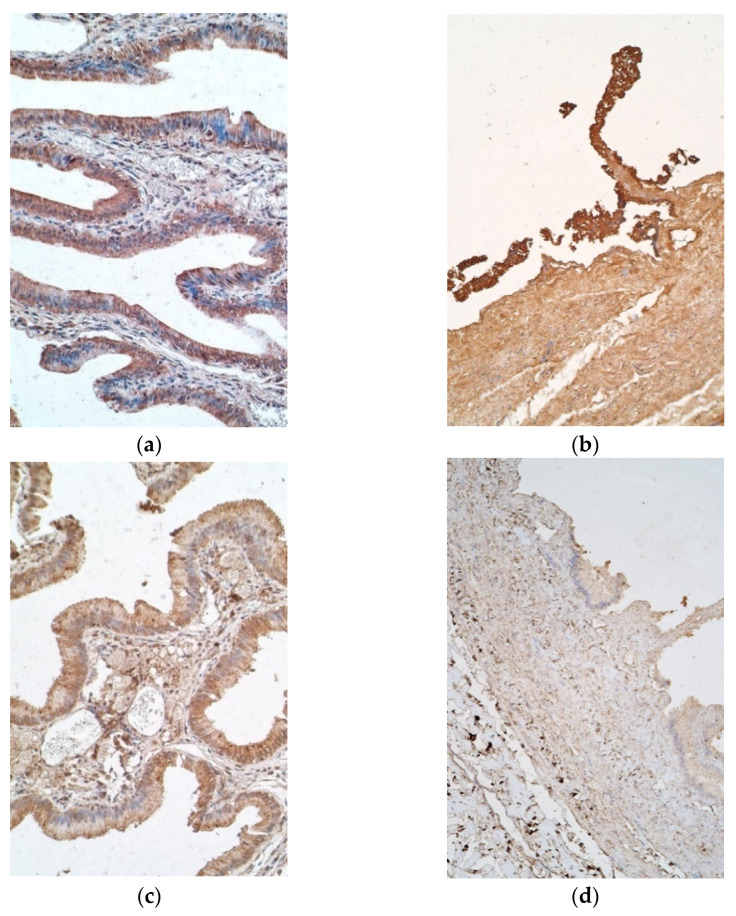
Micrographs of gallbladder in children with cholecystitis and in controls. (**a**) Note numerous IL-7 positive epitheliocytes and moderate number of IL-7 positive cells in connective tissue. IL-7 IMH, ×200; (**b**) note numerous IL-7 positive cells only in the epithelium of control tissue. IL-7 IMH, ×200; (**c**) few to moderate number of IL-8 positive epithelial and connective tissue cells of the patient. IL-8 IMH, ×200; (**d**) control tissue containing only few IL-8 positive cells. IL-8 IMH, ×200.

Interestingly, IL-7 positive cells varied from moderate to numerous in epithelium of patient samples, with six exceptions in which there were few to moderate positive cells, and a similar finding was observed in the controls (Figure 3a). IL-7 stained a moderate number of positive cells in patient connective tissues, whereas control samples contained mostly none, with one sample having a scant number (Figure 3b).

IL-8 positive epitheliocytes varied highly in patient samples, more frequently having none to few positive cells, while controls mostly showed moderate to numerous numbers of positive epitheliocytes, with only one sample containing none. Additionally, connective tissue of patients possessed few to moderate numbers of positive cells, whilst the controls possessed only a few (Figure 3c,d).

**Figure 4:**
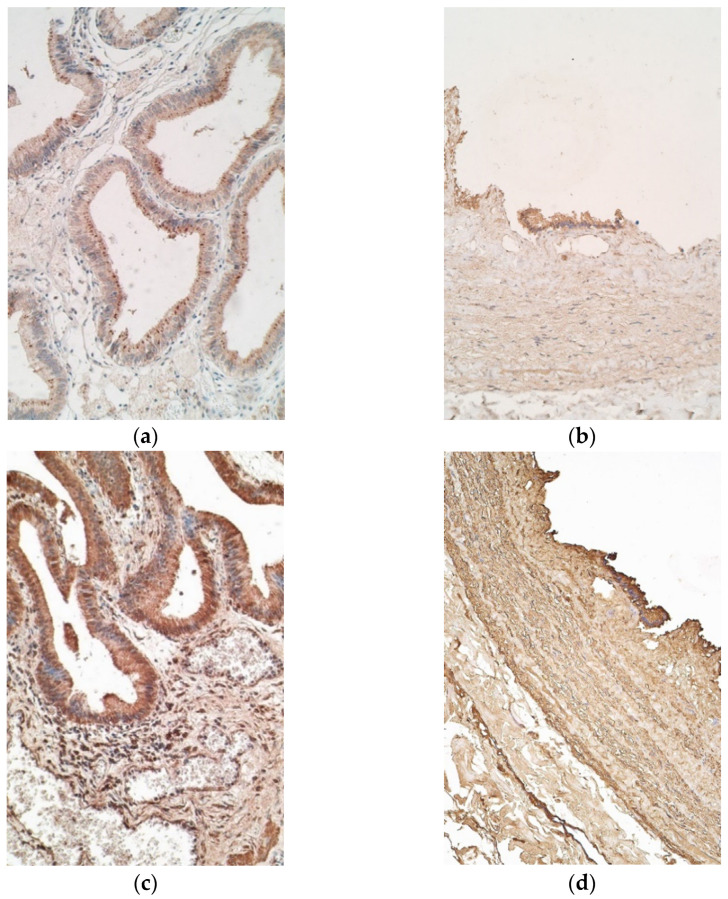
Micrographs of gallbladder in children with cholecystitis and in controls. (**a**) Few to moderate number of IL-10 positive epitheliocytes with absence of the factor in the connective tissue. IL-10 IMH, ×200; (**b**) few of IL-10 positive cells in the epithelium of control sample. IL-10 IMH, ×200; (**c**) numerous positive cells in the epithelium of the patient. IL-17A IMH, ×200; (**d**) epithelium of a control sample, containing numerous IL-17A positive cells. IL-17A IMH, ×200.

Il-10 marked few to moderate positive cells in the epithelium of patients, while controls demonstrated few samples with numerous positive cells. The appearance of IL-10 positive cells in connective tissue was similar in patient and control samples, both containing none to few positive cells (Figure 4a,b).

IL-17A stained moderate to numerous positive cells in patient epithelium and a moderate number of positive cells in connective tissue (Figure 4c). Controls showed numerous IL-17A positive epitheliocytes and only few IL-17A positive connective tissue cells (Figure 4d).

Mann–Whitney U test revealed that statistically significant differences between patient and control samples were seen in IL-1α and IL-17A containing epitheliocytes and also IL-1α, IL-4, IL-6, IL-7, IL-8, and IL-17A positive cells in connective tissues (Table 3). 

A strong positive correlation in patient tissues was detected between IL-1α in epithelium and IL-1α in connective tissue, IL-6 and IL-7, IL-6 and IL-17A, IL-7 and IL-10, and IL-7 and IL-17A in the epithelium. Additional strong positive correlation was detected between IL-6 and IL-7, as well as IL-7 and IL-10 in connective tissue of our patients. Moderate positive correlation was detected between epithelial IL-1α and IL-10, IL-10 and IL-17A, as well as between epithelial IL-6 and IL-4 in connective tissue. IL-6 and IL-4 also correlated moderately in the connective tissue of our patients (Table 4).

## 4. Discussion

The epithelium of gallbladder has many purposes—not only is it a barrier between bile acids and mucosa of the gallbladder, but it also absorbs fluids and secretes H_2_CO_3_ and mucins, providing cytoprotection to cells in contact with bile acids [30]. The interleukins that were found in a highest number in the epithelium of our patient tissues, were IL-1α, IL-4, IL-6, IL-7 and IL-17A, but only IL-1α and IL-17A showed a statistically significant difference between patient and control tissues, suggesting their more significant role in gallbladder in comparison to other cytokines. 

IL-1α is a cytokine that is secreted not only by immune cells, but also by epithelium and endothelium. IL-1α synthesis can be induced by cell stress, injury, infection and proinflammatory mediators. If epithelial cells lack necrosis, they can express IL-1α on their membranes. Gallstones can not only induce cellular stress, but they can also disturb the epithelial barrier, releasing epitheliocyte contents in the extracellular space, thus inducing TNFα and IL-6 expression in immune cells, as well as acute phase protein synthesis by binding to hepatocytes. Therefore, IL-1α expression is linked to beginning and sustaining an immune response in calculous cholecystitis [31]. The fact that IL-1α was in a lower number of positive cells in control tissue epithelium of our patients, proves that healthy tissues do produce this interleukin in basal concentration.

IL-17A was shown in moderate to numerous number of epithelial and connective tissue cells in our patient gallbladders affected by calculous cholecystitis. Out of all IL-17 family members, IL-17C is the only interleukin, which is described to be expressed predominantly by epithelial cells up to now [26]. Interestingly, the synthesis of IL-17C in epithelium can be activated using inflammatory cytokines, such as TNFα and IL-1β or by other IL-17 family members, such as IL-17A/F, what probably might take a place in our patients. On the contrary, IL-17C targets Th17 cells to promote IL-17A/F synthesis, thus potentiating their effects. IL-17A also induces inflammatory gene and antimicrobial peptide expression in the epithelium of the gallbladder, especially in the early stages of the inflammation, showing innate-like cytokine properties [32]. Opposite to expression in epithelium of our healthy children gallbladder, connective tissue showed much indistinct expression of IL-17A in comparison with the patients. Thus, we suggest about the more significant role of IL-17A in the epithelium with significantly high level of it also in the connective tissue of healthy/diseased gallbladder what seems to be novel and intriguing fact and moves the persistently stable high immune response in connective tissue in basic level.

Our study detected a strong correlation between IL-1α in epithelium and in connective tissue of patients, which could be explained by a widespread tissue damage or by intracellular signalling. IL-1α expressed on plasma membranes of epitheliocytes can activate macrophages and neighbouring epithelial cells, stimulating expression of cytokines, which later will recruit migrating primordial myeloid cells that can produce Il-1α in connective tissues. This can cause an inflammatory loop. Due to epithelial cell necrosis, the same loop can be caused by a passive leak of IL-1α from epithelium to the surrounding tissue [14]. 

A strong correlation was also noted between epithelial IL-17A and IL-6. It has been previously proven that IL-17A can heighten other pro-inflammatory cytokine expressions through the activation of NF-κB and MAPKs cascades, and it is possible that in our patients IL-17A induces IL-6 expression in the epithelium, in order to potentiate the immune response to gallstone induced epithelial damage [32]. Another study that researched the IL-6 distribution after bowel injury proved that it is needed for epithelial proliferation after injury; thus, that could be one of the IL-6 effects in our patients with calculous cholecystitis [33].

IL-6 and IL-7 also showed a strong correlation both in epithelium and in connective tissues of our patients. Both of these interleukins promote T cell proliferation and survival, thus working synergistically in order to provide an adequate immune response to the tissue injury by gallstones. In fact, it has been previously shown that IL-6 actually can upregulate the expression of IL-7 receptors to provide an efficient stimulation of T cells [34].

The last strong correlation between interleukins in patient epithelium was between IL-7 and IL-17A. One of IL-7 effects is maintaining T lymphocyte survival in peripheral tissues. A study in 2014 made by Webster et al. has proved that IL-7 is required for IL-17 natural killer cell and CD4+ Th17 cell survival and haemostasis [35]. Additionally, in our study, it could be that mucosal IL-17A positive cells, which survival depends on IL-7, stimulate epithelial cells to produce IL-17A in order to induce other pro-inflammatory cytokine expression at the barrier tissue. 

IL-7 also strongly correlates with IL-10 not only in our patient sample epithelium, but also in connective tissue. This finding is associated with lymphocyte development stimulating effects of these interleukins. IL-7 boosts the survival of naïve and memory T cells, but IL-10 is involved in promoting B Ly survival [21,24]. 

IL-10 and IL-17A appearance moderately correlated in the epithelium of our patients. IL-10 is one of the main negative regulators of Th17 cells and IL-17 producing macrophages [36], yet there is no information about IL-10 inhibitory effects on IL-17A producing epitheliocytes that were observed in our patients. It could be that IL-10 suppresses IL-17A expression in the connective tissues, but not in epithelium, thus both interleukins expression might remain high, and IL-10 effects are not sufficient to downregulate the inflammation in calculous cholecystitis cases. The same can be seen in the moderate correlation between IL-10 and IL-1α in epithelium—IL-10 can inhibit macrophage activation in the connective tissues of our patients, but it does not directly inhibit IL-1α expression in the epithelium, thus IL-1α is still being released from epithelial cells, which in return stimulates epithelial IL-10 production [37].

Mucosa of the gallbladder contains connective tissue with fibroblasts and collagen fibres, as well as immune cells. It is an inflammation site after the epithelial barrier gets destructed by either pathogens or direct injury like gallstones in the case of cholecystitis. The most expressed interleukins in connective tissues of our patients were IL-7, IL-6, IL-4, IL-1α, and IL-17A, all showing a statistically significant difference between patient and control samples. This finding indicates that these interleukins are most important regulators of molecular processes in connective tissue of our patients that have calculous cholecystitis affected gallbladder wall. 

IL-1α is expressed in basal level in epithelium during normal physiological state, and even more during inflammation; thus, its increased expression in connective tissues could also probably be potentiated by epithelial necrosis, caused by gallstone induced injury. This reflects with the idea of other authors, that the reason for an increase in IL-1α positive cells in the connective tissue might be cell signalling induced by epithelial injury [14].

IL-4, an interleukin mostly secreted by immune cells, also basophils, neutrophils and activated Th2, serves a diverse purpose in cholecystitis—it is responsible for T cell differentiation in Th2, proliferation of various tissues, including smooth muscle cells, endothelium and neurons. However, contrary to that, one of its functions is inhibition of pro-inflammatory cytokines (IL-1, IL-6 and TNF-α) [7,16]. Low levels of IL-4 are a predisposing factor to gallstone formation. A study made by Issa and Muthena showed that IL-4 indeed is in a decreased concentration in patients with cholelithiasis [7]. All our patient samples are affected by gallstone induced cholecystitis, thus, the inflammation itself could be the reason of elevated IL-4 in the tissues, and it could work as a negative regulator of inflammation.

IL-6 is a cytokine that, just as with IL-1α, is produced by a variety of cells, including fibroblasts, endothelial and immune cells [18]. Reaction can be induced by exogenous pathogens, through direct stimulation of pattern recognition receptors (PPR’s) such as Toll like receptors. In the case of calculous cholecystitis, epithelial tissue injury can cause a release of damage associated molecular patterns (DAMPs), that can stimulate the same PPR’s and induce pro-inflammatory cytokine, like IL-6 release. IL-1α has been proven to be a nuclear DAMP, and we have demonstrated that all of the patient tissues contained IL-1α positive cells both in epithelium and connective tissue [38]. As IL-6 role in cholecystitis is stimulation of acute phase protein and albumin synthesis, as well as stimulation of B cells and immunoglobulin production [19], we speculate that IL-6 is a key interleukin of gallstone wall response to the inflammatory process.

Although, it has been previously proven that IL-7 is expressed by epithelial cells [21] our research showed a statistically significant increase in IL-7 positive cells in connective tissue. There IL-7 can be produced also by fibroblasts in bone marrow and dendritic cells [39]. There are a scant number of studies about this interleukin’s high expression in the gallbladder during cholecystitis [9]. Our study has proven that IL-7 is expressed in connective tissue of patient gallbladder affected by calculous cholecystitis in a statistically significant number of cells. Probably the function of IL-7 could be boosting the survival of naïve and memory T cells, thus sustaining the necessary inflammatory reaction, through Blc2 induced proliferation of T cells [40].

In our study, epithelial IL-6 moderately correlated with IL-4 in connective tissues of our patients. The possible explanation for this could be that IL-6 produced by epithelial cells can bind to IL-6 receptors on CD4+ cells, stimulating them to release IL-4, supporting CD4+ T cell differentiation into Th2, which further will stimulate B cell proliferation and antibody synthesis, that is necessary in calculous cholecystitis [41].

Our study has some limitations, one of which is the relatively small number of controls which could influence our results. However, due to the ethical concerns, these tissues are very hard to obtain, which also limits the chances of children and adult gallbladder tissue comparison. Furthermore, in our study we only used immunohistochemistry to evaluate the presence of interleukins in the tissue samples. It is possible that if we had implied other methods, such as ELISA, we may have been able to examine the data more accurately. Another valuable procedure would be identification of genes that are involved in morphopathogenesis of calculous cholecystitis. Therefore, we are planning to continue this study and involve some of the above mentioned issues in future research.

## 5. Conclusions

The increase of IL-1α, IL-4, IL-6, IL-7, IL-8 and IL-17A positive cells suggest the role of these interleukins in the morphopathogenesis of the calculous cholecystitis. 

The epithelial distribution of IL-1α and IL-17A proves their selective role in the inflammation and immune system activation in the border tissue. Correlations, especially the ones between epithelial IL-17A and IL-6, IL-7, and IL-10 prove the epithelial barrier function and induction of immune function in underlying connective tissue. 

The variable appearance of IL-1α, IL-4, IL-6, IL-7, IL-8 and IL-17A positive cells in connective tissue proves the multifunctional interleukin role there. The correlations between IL-4 and IL-6, as well as between IL-6 and IL-7, suggest that the initiated inflammatory response to gallstone induced injury is being actively intensified and sustained through cytokines modulated intercellular signalling pathway.

## Figures and Tables

**Table 1:** Number of interleukin positive cells in the gallbladder epithelium and connective tissue of patient samples.

IL’s/ Patients	IL-1α		IL-4		IL-6		IL-7		IL-8		IL-10		IL-17A	
	E	CT	E	CT	E	CT	E	CT	E	CT	E	CT	E	CT
**1.**	++	+	++	++	++	++	++/+++	++	0	0/+	+/++	0/+	+++	++/+++
**2.**	+/++	+	+++	++	++	++	++/+++	++	0/+	+	+/++	0	+/++	+/++
**3.**	++	+	No	No	+/++	++	+/++	++/+++	+/++	+	++/+++	0/+	++	++
**4.**	+++	++	+++	++/+++	++/+++	++/+++	++	++/+++	0	+/++	++	+	++	+/++
**5.**	N	++	N	++/+++	N	++	N	++/+++	N	0/+	N	0/+	N	++/+++
**6.**	N	+/++	N	++	N	++	N	++/+++	N	+	N	0/+	N	++
**7.**	++/+++	+/++	++/+++	++/+++	+++	++/+++	+++	++/+++	0	++	++	0/+	+++	+/++
**8.**	++/+++	+/++	++	+/++	++/+++	+/++	+++	++	+++	+/++	++/+++	0	+++	+
**9.**	+/++	+	+/++	++	++	++	+/++	++	0	+/++	+	0	+/++	+/++
**10.**	++	+	++/+++	++/+++	+++	++	++/+++	++	++/+++	+/++	+/++	0	++/+++	++
**11.**	+/++	+	+++	++	+/++	++	+/++	+/++	+/++	+/++	0/+	0	+/++	+/++
**12.**	+/++	+	+/++	++	++	+/++	+/++	+/++	+/++	+/++	0/+	0	+/++	+/++
**13.**	+/++	+	++/+++	++	++/+++	++	++	++/+++	++/+++	+/++	++	0/+	++/+++	++
**14.**	+/++	+	++	++/+++	++/+++	+++	+/++	+++	++/+++	++	0/+	0/+	++/+++	++/+++
**15.**	+	+/++	++	++	++	+++	++	++/+++	0	0/+	+	0	++	++/+++
**16.**	++	+/++	+++	++	+++	++/+++	++/+++	++/+++	0/+	+/++	++	+	++/+++	++/+++
**17.**	++/+++	+/++	+++	+/++	+/++	++	+/++	++	+	+/++	0/+	0/+	+/++	++/+++
**18.**	++/+++	+/++	+++	++	+++	+/++	+++	+/++	+++	+/++	++	0	+++	++
**19.**	+	+	++/+++	++/+++	++/+++	++/+++	++	++	0/+	0/+	+	0	++/+++	++
**20.**	N	+	N	++	N	++/+++	N	++/+++	N	+	N	0	N	++
**Avg**	++	+/++	++/+++	++	++/+++	++	++/+++	++	0/+	+/++	+/++	0/+	+++	++

Abbreviations: IL’s—Interleukins; IL-1α—interleukin 1α; IL-4—interleukin 4; IL-6—interleukin 6; IL-7—interleukin 7; IL-8—interleukin 8; IL-10—interleukin 10; IL-17A—interleukin 17A; E—epithelium; CT—connective tissue; N—no epithelium; No—no tissue sample; Avg—average; 0—no positive cells, 00/+—a scant number of positive cells, 0/+—occasional positive cells, +—few positive cells, +/++—few to moderate number of positive cells, ++—moderate number of positive cells, ++/+++—moderate to numerous positive cells, +++—numerous positive cells in the visual field.

**Table 2:** Number of interleukin positive cells in the gallbladder epithelium and connective tissue of control samples.

IL’s/ Controls	IL-1α		IL-4		IL-6		IL-7		IL-8		IL-10		IL-17A	
	E	CT	E	CT	E	CT	E	CT	E	CT	E	CT	E	CT
**1.**	N	0/+	N	+	+	+	+++	0	++	+	00/+	0	+++	+
**2.**	0	0	+++	+	+++	+	+++	0	+++	0	+++	0	+++	+
**3.**	0	0	+++	0	+++	0/+	+++	0	+++	0	++	0	+++	+
**4.**	N	00/+	N	+	N	+	+++	+	N	+	0/+	0/+	+++	+/++
**5.**	0	0	+++	+	+++	+	++	00/+	0	0	+++	0	+++	+
**6.**	N	0	N	+	No	No	++	0	N	+	+++	+	+++	+
**7.**	N	0	N	+	N	+	N	0	N	+	N	0/+	+++	+
**Avg**	0	0	+++	+	+++	+	++/+++	0	++/+++	+	++	0/+	+++	+

Abbreviations: IL’s—Interleukins; IL-1α—interleukin 1α; IL-4—interleukin 4; IL-6—interleukin 6; IL-7—interleukin 7; IL-8—interleukin 8; IL-10—interleukin 10; IL-17A—interleukin 17A; E—epithelium; CT—connective tissue; N—no epithelium; No—no tissue sample; Avg—average; 0—no positive cells, 00/+—a scant number of positive cells, 0/+—occasional positive cells, +—few positive cells, +/++—few to moderate number of positive cells, ++—moderate number of positive cells, ++/+++—moderate to numerous positive cells, +++—numerous positive cells in the visual field.

**Table 3:** Significant differences in interleukins of epithelium and connective tissue between patient and control tissue samples.

Interleukins	IL-1α E	IL-1α CT	IL-4 CT	IL-6 CT	IL-7 CT	IL-8 CT	IL-17A E	IL-17A CT
***p*** **-Value**	<0.001	<0.001	<0.001	<0.001	<0.001	0.008	0.002	<0.001
**Mean**	1.8824	1.2750	2.105	2.150	2.2000	1.250	2.235	1.950
**St. Deviation**	0.57362	0.34317	0.3153	0.4323	0.41039	0.4730	0.5894	0.4560

Abbreviations: IL-1α—interleukin 1 α; IL-4—interleukin 4; IL-6—interleukin 6; IL-7—interleukin 7; IL-8—interleukin 8; IL-17A—interleukin 17A; E—epithelium; CT—connective tissue.

**Table 4:** Spearman’s rank correlation coefficient revealing correlations between different interleukins in connective tissue and epithelium.

Factor 1	Factor 2	R	*p*-Value
**Strong Positive Correlation (0.60–0.79)**			
IL-1α E	IL-1α CT	0.643	0.005
IL-6 E	IL-7 E	0.699	0.002
IL-6CT	IL-7 CT	0.692	0.001
IL-7E	IL-10 E	0.625	0.007
Il-7CT	IL-10 CT	0.63	0.003
IL-6E	IL-17A E	0.709	0.001
IL-7E	IL-17A E	0.749	0.001
**Moderate Positive Correlation (0.40–0.59)**			
IL-1α E	IL-10 E	0.543	0.024
IL-4 CT	IL-6 E	0.501	0.048
IL-4CT	IL-6 CT	0.492	0.032
IL-10 E	IL-17A E	0.561	0.019

Abbreviations: IL-1α—interleukin 1 α; IL-4—interleukin 4; IL-6—interleukin 6; IL-7—interleukin 7; IL-8—interleukin 8; IL-17A—interleukin 17A; E—epithelium; CT—connective tissue.

## Data Availability

All datasets used/analysed in the present study are presented in the result sections of the manuscript.
